# Applicability of an immersive virtual reality system to assess egocentric orientation of older adults

**DOI:** 10.1055/s-0042-1759762

**Published:** 2023-03-14

**Authors:** Juliana Magalhães da Silva, Michelle Didone dos Santos, Raquel Quimas Molina da Costa, Emerson Galves Moretto, Larissa Alamino Pereira de Viveiro, Roseli de Deus Lopes, Sonia Maria Dozzi Brucki, José Eduardo Pompeu

**Affiliations:** 1 Universidade de São Paulo, Departamento de Fisioterapia, Faculdade de Medicina, Fonoaudiologia e Terapia Ocupacional, São Paulo SP, Brazil.; 2 Universidade de São Paulo, Departamento de Neurologia, Faculdade de Medicina, São Paulo SP, Brazil.; 3 Universidade de São Paulo, Departamento de Engenharia de Sistemas Eletrônicos, Escola Politécnica, São Paulo SP, Brazil.

**Keywords:** Orientation, Spatial, Virtual Reality, Aged, Reproducibility of Results, Data Accuracy, Orientação Espacial, Realidade Virtual, Idoso, Reprodutibilidade dos Testes, Confiabilidade dos Dados

## Abstract

**Background** Spatial orientation is a cognitive domain frequently compromised in patients with Alzheimer disease (AD) and may be one of its first clinical manifestations. Some studies have shown that allocentric integration with egocentric spatial information seems to be impaired in this pathology. There is no consensus on how best to assess spatial orientation and traditional tests lack ecological validity, but, recently, virtual reality (VR) has provided new opportunities for this assessment.

**Objectives** To analyze the applicability and stability of an immersive virtual task developed to assess spatial orientation, the Spatial Orientation in Immersive Virtual Environment Maze Test (SOIVET-Maze) in older adults with and without mild cognitive impairment.

**Methods** Forty-three older adults were included in the study, 24 without cognitive impairment and 19 with mild cognitive impairment. Applicability was assessed by the Witmer and Singer Sense of Presence Questionnaire and a questionnaire for adverse events of cybersickness. To assess stability, participants were assessed twice with an interval of 7 to 14 days, and the intraclass correlation coefficient was calculated between visits. The t test or the Mann-Whitney test was used to compare applicability and stability between groups.

**Results** There was no significant difference between the groups regarding applicability. A strong correlation between the first and second day of testing was found in the mild cognitive impairment group.

**Conclusion** The SOIVET-Maze task showed excellent applicability and good stability, favoring its clinical application for the evaluation of spatial orientation in older adults.

## Resumo

**Antecedentes** A orientação espacial é um domínio cognitivo frequentemente comprometido em pacientes com doença de Alzheimer (DA) e pode ser uma das suas primeiras manifestações clínicas. Alguns estudos demonstraram que a integração alocêntrica com informações espaciais egocêntricas parece prejudicada nessa patologia. Não há um consenso sobre qual a melhor forma de avaliar a orientação espacial e os testes tradicionais carecem de validade ecológica; porém, recentemente, a realidade virtual (RV) proporcionou novas oportunidades para esta avaliação.

**Objetivos** Analisar a aplicabilidade e estabilidade de uma tarefa virtual imersiva desenvolvida para avaliar a orientação espacial, o Spatial Orientation in Immersive Virtual Environment Maze Test (SOIVET-Maze) em idosos com e sem comprometimento cognitivo leve.

**Métodos** Quarenta e três idosos foram incluídos no estudo, 24 sem comprometimento cognitivo e 19 com comprometimento cognitivo leve. A aplicabilidade foi avaliada pelo Witmer and Singer Sense of Presence Questionnaire e um questionário para eventos adversos de *cybersickness* . Para avaliar a estabilidade, os participantes foram avaliados 2 vezes com intervalo de 7 a 14 dias, e o coeficiente de correlação intraclasse foi calculado entre as visitas. O teste t ou o teste de *Mann-Whitney* foi utilizado para comparar a aplicabilidade e estabilidade entre os grupos.

**Resultados** Não houve diferença significativa entre os grupos quanto à aplicabilidade. Uma forte correlação entre o primeiro e o segundo dia de teste foi encontrada no grupo de comprometimento cognitivo leve.

**Conclusão** A tarefa SOIVET-Maze apresentou excelente aplicabilidade e boa estabilidade, favorecendo sua aplicação clínica para avaliação da orientação espacial em idosos.

## INTRODUCTION

Spatial orientation is the ability to locate oneself in the surroundings and it is an essential skill for individual autonomy and participation in society. 1 It is a complex cognitive function that involves other cognitive processes, such as attention and memory, as well as sensory information that allows individuals to locate themselves in the environment. 2

During navigation, spatial orientation can be divided between egocentric and allocentric orientation. 3 Egocentric orientation is modulated by the parietal lobes and subcortical regions and refers to the ability to locate oneself through the body position in the environment. 4 In contrast, allocentric orientation is the ability to locate oneself using topographic landmarks, that is, reference points in the environment and is modulated by the hippocampus. 4 5

Spatial disorientation occurs when the individual begins to have difficulty in recognizing familiar routes, learning new routes or locate themselves in the environment. 4 It is an early manifestation of Alzheimer disease (AD) cognitive decline and may also be present in mild cognitive impairment (MCI). 6

A careful evaluation is necessary to identify spatial disorientation; the earlier this impairment is detected, the greater is the possibility of identifying initial pathological cognitive decline. 7 8 However, the current literature lacks a reference standard test for assessing spatial orientation, and traditional paper and pencil tests are not ecological or sensitive enough to identify spatial disorientation. 8 9 Ecological assessments try to reproduce real-world impairments as close as possible and can be performed in real or virtual environments, on larger scales. 6

One of the conventional tests used in clinical assessment is the Money Road Map Test (MRMT). 10 The test aims to assess right-left orientation, and it composed of a two-dimensional aerial representation of a map on paper, like a city seen from above. It presents a tortuous route, marked by 32 left and right turns at different angles. 11 For this test, participants verbally describe the line route, and inform whether it went to the right or to the left at each curve. The MRMT was adapted to a computer task by Morganti et. al. 12 and was able to differentiate spatial orientation performance between older adults without cognitive impairment and early stages Alzheimer's disease. 12

Virtual Reality (VR), defined as a computer-generated technology that promotes interaction between users and virtual environments, has been increasingly used in healthcare. 13 Tasks performed in virtual environments use high-intensity stimulation provided by increased sensory, visual, and auditory feedback. 13 In the last decades, VR has proven to be an effective instrument for the assessment of cognitive functions such as attention, memory, and executive functions. 1

In immersive virtual reality (IVR), the individual is transported into the virtual world, that is, it corresponds to the immersion term attributed to the feeling of being present in the virtual environment. Immersive virtual reality allows users to concentrate more on the task and to use more natural and realistic motor and sensory patterns, thus favoring the validity of the evaluation. 14 However, this type of interaction can cause adverse effects, such as cybersickness, characterized by the onset of vestibular and autonomic symptoms – such as motion sickness, dizziness, and nausea, among others – which limits its comprehensive use in clinical practice. 15

Given the advantages of IVR for cognitive evaluation, our group developed a VR system that assesses the spatial orientation of older adults. The Spatial Orientation Test in an Immersive Virtual Environment (SOIVET) system is divided into two tasks: the SOIVET-Maze task and the SOIVET-Route task. The immersive characteristic of the system increases the sense of presence, depending on the ability of this technology to stimulate sensory perceptions, through visual, vestibular, and somatosensory receptors, also activating brain areas closely related to real life. Following the first evidence of applicability and tolerability in younger adults, 16 few modifications were made in the system, allowing for its use in older adults. The SOIVET system demonstrated to be a valid tool for the assessment of spatial orientation in older adults with and without MCI. 17 In particular, the MAZE task, based on the MRMT, evaluates the transposition between a primarily allocentric view (top view of the map) to an egocentric one (first person view), allowing the individual to navigate in the environment and experience changes in direction and the environment around at each turn. 12 17 However, no psychometric properties such as applicability and stability were reported for the SOIVET-Maze task.

In view of the lack of consensus on a reference standard to assess spatial orientation and the advantages of evaluating it by means of tasks as close as possible to the way spatial orientation is recruited on a daily basis, the present study aims to analyze the applicability and stability of an immersive task developed to evaluate spatial orientation (SOIVET-Maze) in older adults with and without mild cognitive impairment (MCI).

## METHODS

### Sample

The study was conducted in the city of São Paulo, state of São Paulo, Brazil, at the Interdisciplinary Center for Interactive Technologies (Centro Interdisciplinar de Tecnologias Interativas [CITI]) of the Polytechnic School of Electrical Engineering of the Universidade de São Paulo (Escola Politécnica de Engenharia Elétrica da Universidade de São Paulo). All participants who agreed to participate in the research signed the free and informed consent term approved by the Ethics Committee of the Hospital das Clinicas, School of Medicine of the Universidade de São Paulo (process n° 11017019.1.0000.0065).

Healthy older adults: 24 elderly residents of the community considered without cognitive impairment because they did not present a complaint or objective evidence, but only a verification through the screening score with the Addenbrooke Cognitive Examination, with scores > 82 points. – Revised (ACE-R). 18 19

Older adults with MCI: 19 participants with a medical diagnosis of MCI recruited from the Reference Center for Cognitive Disorders (Centro de Referência de Distúrbios Cognitivos [CEREDIC]), Hospital das Clinicas, School of Medicine of the Universidade de São Paulo (Hospital das Clínicas, Faculdade de Medicina da Universidade de São Paulo [HCFMUSP]).

Eligible participants were of both sexes, ≥ 60 years old, did not present motor or neurological limitations that would prevent test performance, presented normal or corrected visual and auditory acuity, and did not have a history of vertigo or labyrinth dysfunction. Participants who experienced adverse effects that prevented the continuation of the task were excluded from the study.

### Sociodemographic and clinical characteristics of the sample

For sociodemographic characterization, a medical and demographic questionnaire was applied including personal information such as age, sex, and education, as well as comorbidities. A questionnaire regarding familiarity with technology was also applied to identify the profile of the users and investigate potential impacts on the understanding and performance of the test. All questionnaires used to characterize the sample were developed by our research group. 16

### Procedures

The assessments were divided into two evaluation days. On the first day, participants were screened using the ACE-R and a motion sickness screening questionnaire. Subsequently, the sociodemographic and medical screening questionnaires, the familiarity with technology questionnaire, and the traditional MRMT were applied.

### Money road map test

The MRMT is a neuropsychological test of right-left discrimination, which requires egocentric mental rotation in space, in which the participant must verbally describe the dotted path on the map. 16 20

During the evaluation, participants cannot turn their heads or manipulate the paper to make such decisions, being supervised by a trained evaluator who remains beside the individual, noting possible errors and successes, but without influence on the test. Upon completion of the test, participants move to the next phase of the assessment.

### The SOIVET-Maze task

**Figure 1:**
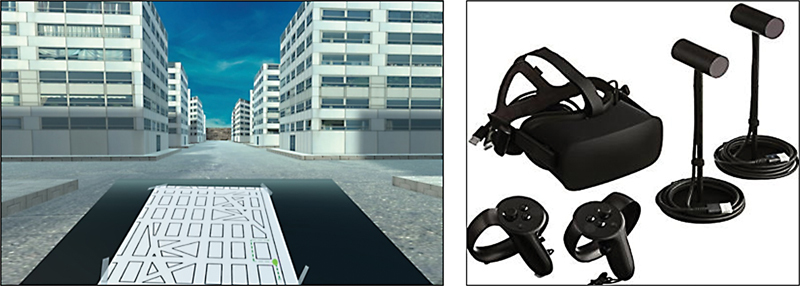
Test representation in the immersive environment of SOIVET-Maze and Oculus Rift model CV1, with both touch controls and sensors.

Participants were asked to perform the SOIVET-Maze task using a VR system composed of a head-mounted display Oculus Rift model CV1 synchronized to 2 touch controls ( Figure 1 ), as well as an Intel i7 notebook with 16 GB of memory and NVIDIA GeForce GTX 980M video card. The task was projected on the lenses of the headset and the individual navigated the environment using manual controls 21 in addition to exploring the environment by moving their head to the right and left. A familiarity with the system was created before for participants to understand and learn how to navigate in the environment (training stage). They navigated using first person perspective, inside a labyrinth of buildings with identical walls, gray floors and a blue sky, and an image of the labyrinth map with a path identified by a dotted line represented at the bottom of the screen ( Figure 1 ).

Participants were evaluated using by the original scoring system of the MRMT: the total number of correct turns made at in each decision to take a right or left turn. This scoring system was used, both in the traditional task evaluated on paper, and in the virtual task. The task was projected at the same time in the virtual reality and on the computer screen, which allowed the evaluator to follow their entire route.

The SOIVET-Maze task was carried out in two stages:

- First stage – Task: Participants performed a reduced version of the SOIVET-Maze task, in which the maze map presented only the first curves of the original path, running a maximum of two times to create familiarity with the system and the task.
- Second stage - Complete Task: Participants were invited to perform the complete task, in which only two attempts at success were allowed. Upon the third mistake, the test ended automatically. Only the values of the second stage were used for statistical analysis. During the task, a trained investigator stood next to participants, following their progress through the computer monitor, nevertheless. The performance of the participants was not influenced by this observation, and scores were calculated automatically by the system.

**Figure 2:**
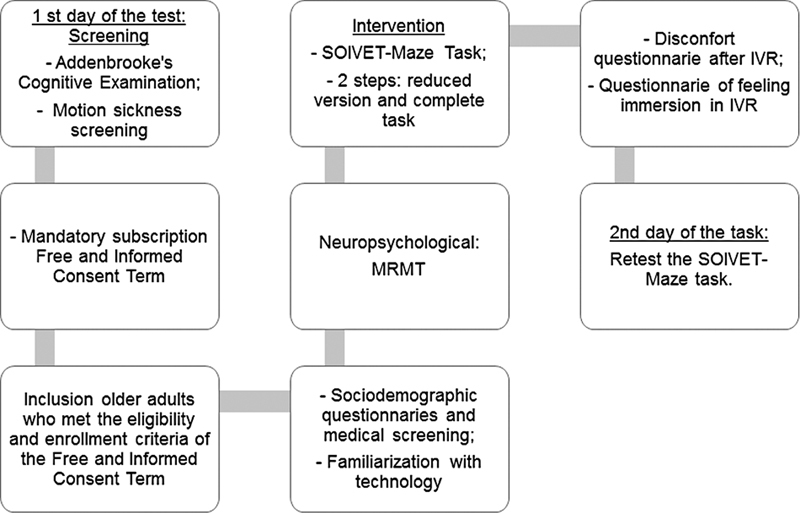
Sequence of procedures performed in the present study.

Finally, on the 2 ^nd^ day of assessment, after an interval of 7 to 14 days, participants were invited to the SOIVET-Maze task once more (retest). The sequence of procedures performed in the present study is described in Figure 2 .

### Assessment applicability and stability

To assess applicability, the Cybersickness Questionnaire was used. This instrument was translated and adapted to the Portuguese language by Carvalho et al, 22 being adapted by our research group for cybersickness symptoms. The aim was to track the symptoms of cybersickness on a four-point Likert scale. It contains 16 questions in total; 1 point is assigned to each affirmative answer, so that the maximum score is 64 points.

The Witmer and Singer Presence Questionnaire was also used to assess applicability. 23 It consists of 22 self-reported items; each item is composed of a 7-point Likert scale, regarding the experience with the virtual environment. Participants describe the experience within the virtual environment by placing a cross on the scale. The maximum score is 154 points, and the higher the score, the greater the sense of immersion.

To assess stability, the SOIVET-Maze task was applied with an interval of 7 to 14 days.

### Statistical analysis

The data collected were tabulated on Microsoft Excel (Microsoft Corp., Redmond, WA, USA) and analyzed on JASP 0.16. The clinical and sociodemographic characteristics were presented using the values of means, standard deviations (SDs), medians, and interquartile ranges.

For the analysis of tolerability and sense of presence and immersion, the Mann-Whitney test was applied. For test-retest reliability and stability, the calculation of the intraclass correlation coefficient (ICC) was applied, having been carried out between the 1 ^st^ and the 2 ^nd^ day of evaluation. The sample calculation was performed using effect with a power of 80% and alpha of 0.05. An ICC ≥ 0.6 was considered to be clinically relevant. The result of the sample calculation pointed to 15 participants in each group. 14 To interpret the effect size, the strength values of rank-bisserial correlation were considered.

## RESULTS

### Sample characterization

**Figure 3:**
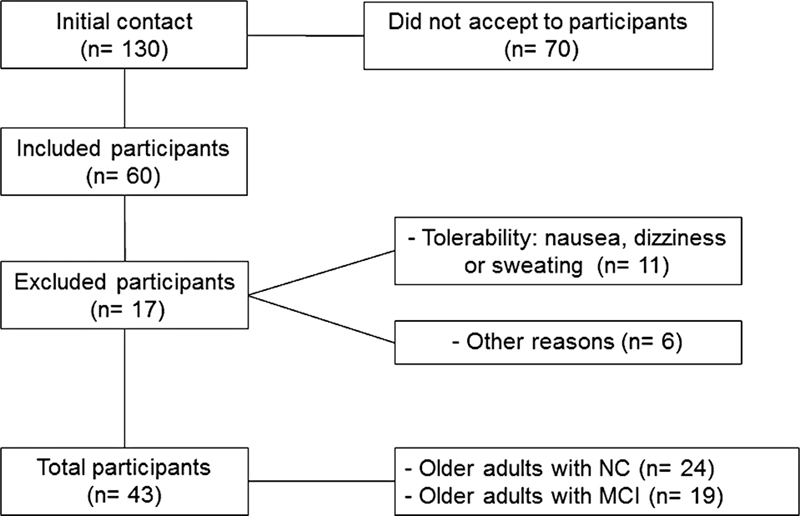
Flowchart of sample participation.

Sixty participants were included; all of them participated in the 1 ^st^ day of assessment. From that moment, 17 participants were excluded—11 for presenting adverse events such as motion sickness, nausea, and/or excessive sweating, and 6 for other reasons, among them giving up on participating alleging difficulties due to the distance from the place, unavoidable commitments, and incompatibility of schedules due to participation in other surveys. Thus, a total of 43 people participated in the study – 24 without MCI and 19 with MCI ( Figure 3 ).

The mean age of the participants was 71.3 (±  5.6) years old, and 62.8% were women. Most of the participants had comorbidities; the most common were hypertension, pre-existing diseases or injuries, diabetes mellitus and osteoporosis. The complete analyses of the sample characterization are described in Table 1 .

**Table 1:** Sociodemographic characterization of the total sample of elderly people and comparison between groups of elderly people without mild cognitive impairment and elderly people with mild cognitive impairment

		Total sample ( *n* = 43)	NC group ( *n* = 24)	MCI group ( *n* = 19)	Appropriate statistical test	*p-value*
Age, mean (SD)		71.3 (5.6)	70.5 (5.6)	72.3 (5.6)	t _(41)_ = -1.025*	0.311
Sex, *n* (%)	Female	27 (62.8)	15 (62.5)	12 (63.2)	x ^2^_(1)_ = 0.002**	0.965
	Male	16 (37.2)	9 (37.5)	7 (36.8)		
Schooling, *n* (%)	1 to 4 years	2 (4.6)	2 (8.3)	0	x ^2^_(3)_ = 11.049**	0.011
	5 to 8 years	12 (27.9)	3 (12.5)	9 (47.4)		
	9 to 12 years	27 (62.8)	19 (79.2)	8 (42.1)		
	Over 12 years	2 (4.7)	0	2 (10.5)		
ACE-R, median (IQR)		92.0 (11.0)	94.5 (6.0)	86.0 (10.0)	W = 364.500***	<0.001
Questionnaire Familiarity with technology, median (IQR)		17.0 (13.0)	19.0 (10.5)	16.0 (6.5)	W = 312.500***	0.039
Comorbidities, *n* (%)	Yes	31 (72.1)	17 (70.8)	14 (73.7)	x ^2^_(1)_ = 0.043**	0.836
	No	12 (27.9)	7 (29.2)	5 (26.3)		

Abbreviations: NC, normal cognitive; ACE-R, Addenbrooke's Cognitive Examination; IQR, interquartile range; MCI, Mild Cognitive Impairment.

Notes: *independent t-test; **chi-squared test; ***Mann-Whitney test. Note: numerical values represented by mean ± standard deviation (SD) and absolute number (%).

### Applicability

There were no differences between groups on cybersickness questionnaire scores (W = 198.000; *p* = 0.439; r _b_ = −0.132) with 0 (±  1.0) and 1.0 (±  2.0) points, respectively for the NC and MCI groups ( Table 2 ).

**Table 2:** Tolerability compared between the groups of older adults without mild cognitive impairment and older adults with mild cognitive impairment

	Total sample ( *n* = 43)	Older adults without MCI ( *n* = 24)	Older adults with MCI ( *n* = 19)	Mann-Whitney test	*p-value*	Effect Size
Prediscomfort, median (IQR)	0 (0.5)	0 (0)	0 (1.0)	W = 189.000	0.219	−0.171
Cybersickness, median (IQR)	1.0 (2.0)	0 (1.0)	1.0 (2.0)	W =198.000	0.439	−0.132

Abbreviations: MCI, Mild Cognitive Impairment.

Notes: Prediscomfort - Prior screening for motion sickness Questionnaire; Effect size (rank-biserial correlation): very high: 0.90–1.00; high: 0.70–0.90; moderate: 0.50–0.70; low: 0.30–0.50; small: 0.10–0.30.

As for the sense of presence, there was no difference between groups (W = 161.000; *p* = 0.104; r _b_ = −0.294), where 131.0 (±  25.5) was found for the participants without MCI and 134.0 (±  21.0) for the participants with MCI ( Table 3 ).

**Table 3:** Sense of presence compared between the groups of older adults without MCI and older adults with MCI

	Total sample (n = 43)	Older adults without MCI (n = 24)	Older adults with MCI (n = 19)	Mann-Whitney test	p value	Effect Size
Presence Questionnaire, median (IQR)	134.0 (23.0)	131.0 (25.5)	134.0 (21.0)	W = 161.000	0.104	−0.294

Abbreviation: MCI, Mild Cognitive Impairment.

Notes: Effect size (rank-biserial correlation): very high: 0.90–1.00; high: 0.70–0.90; moderate: 0.50–0.70; low: 0.30–0.50; small: 0.10–0.30.

### Stability

The intraclass correlation coefficient (ICC) showed a good correlation in the group with MCI, reasonable correlation in the total sample, and weak correlation in the group without MCI ( Table 4 ).

**Table 4:** Intraclass Correlation Coefficient (ICC) between 1st and 2nd day of the SOIVET-Maze test

		1st day - SOIVET- Maze (ICC)	Lower 95% CI	Upper 95% CI
Total sample (n = 43)	2nd day - SOIVET-Maze	0.476	0.255	0.649
NC group (n = 24)	2nd day - SOIVET-Maze	0.288	0.040	0.503
MCI group (n = 17)	2nd day - SOIVET-Maze	0.667	0.499	0.786

Abbreviation: MCI, Mild Cognitive Impairment.

Note: ICC<0.4: weak; 0.40 a 0.60: reasonable; 0.60 a 0.75: good; 0.75 a 1.0: strong.

## DISCUSSION

The present study aimed to analyze the applicability and the stability of the SOIVET-Maze system.

The applicability analysis of the SOIVET-Maze system demonstrated low levels of adverse symptoms in both groups, and no differences were found between the groups, despite a small numerical difference in the group of older adults with MCI. This result shows us in a positive way that all participants had tolerance for the use of VR and can justify that the difference in the MCI group may have been due to individual vulnerability, either by intrinsic factors such as age, gender, or sensory conflicts, by extrinsic factors such as the landscape of the virtual environment, or by emotional factors related to anxiety, causing greater discomfort. A study previously conducted by Costa et al. 16 meets this hypothesis, as it used the same SOIVET system and the Maze task, in which they assessed the applicability to young adults and found a strong correlation between tolerability and prior seasickness scores and high immersion rates and sense of presence.

In addition, participants reported high levels of immersion and presence; such finding is considered essential for the cognitive engagement capacity and for the test to be considered ecological. 17 Our results are in line with a study by Ijaz et al., 24 which evaluated the applicability and usability of an immersive VR platform to assess spatial navigation in older adults. Their results revealed that, despite the relative novelty of VR technology for this population, they made few navigation errors, identified challenging landmarks successfully and presented high levels of presence.

Regarding stability, we had a reasonable correlation in the total sample group of older adults, which means that the test did not undergo significant variation when performed from one day to the next of the evaluation, as well as by different evaluators on the 1 ^st^ and 2 ^nd^ days. Ranjbar Pouya et al. 25 also found positive results in a reliability study correlating the spatial orientation with the cognitive capacity of young and older adults in VR assessment with a repetition interval of ∼ 6 months. Despite the long interval, the study presented strong correlations in all of its analyses, which showed the absence of test learning bias. In the present study, the sample was divided into groups, and we found a good correlation only in the group with MCI, although a lower correlation was found in the normal cognition group. This might have occurred due to the low familiarity of the participants with the system – upon evaluation of the performance of the normal cognitive (NC) group, we noticed that their score increased between the two testing days. The test used was exactly the same as the retest and was applied by trained evaluators, which may explain the results. On the other hand, the SOIVET system automatically scores the performance of each participant, therefore being independent of the interpretation of the evaluators. The participants with MCI did not show improvement in performance, which may be explained by cognitive impairment, 7 as well as demonstrated by our research group. 26 This information is relevant for understanding the pathology of MCI and its ability to learn and perform in virtual tests, because although we have evaluated exactly one task that evaluates an impaired cognitive area in the MCI, it is not possible to establish the exact reason of this discreet improvement from one day to another.

Immersive virtual reality offered the possibility of older adults moving through the environment in a much more realistic way. On the other hand, it presented a much greater difficulty for familiarization, considering that this type of technology is not usual for older adults. In addition, the complexity of the task for requiring mental rotation, 12 that is, having to move around the environment from an egocentric perspective, while maintaining the perception that the body remained in a chair, may have caused a conflict of sensations. One solution to this problem would be to increase the number of trials on the day of the evaluation.

In general, we intend to increase the sample size in the present study to obtain more consistent results and transport the immersive VR task to a mobile device, such as a tablet that has a rotation sensor, which is a possibility to include older adults who present greater sensitivity to motion sickness. Although the sense of presence is possibly smaller when compared to that of an immersive task, the advantages of this device would still be greater, as well as allowing greater access to the immersive system by professionals in their clinical practice.

In conclusion, the SOIVET-Maze task showed excellent tolerability and a high sense of presence, being applicable to older adults with and without MCI. It showed evidence of test-retest stability in the 1 ^st^ and 2 ^nd^ days of evaluation of the total sample and variations in stability were found between the NC and MCI groups, which indicate better performance in the NC group after familiarity with the system.
